# Women with ovarian cancer’s information seeking and avoidance behaviors: an interview study

**DOI:** 10.1093/jamiaopen/ooae011

**Published:** 2024-02-21

**Authors:** Yu Chi, Vivian Hui, Hannah Kunsak, Peter Brusilovsky, Heidi Donovan, Daqing He, Young Ji Lee

**Affiliations:** School of Information Science, College of Communication and Information, University of Kentucky, Lexington, KY 40506, United States; Center for Smart Health, School of Nursing, The Hong Kong Polytechnic University, Hong Kong, China; Department of Health and Community Systems, Health and Community Systems, School of Nursing, University of Pittsburgh, Pittsburgh, PA 15261, United States; Department of Health and Community Systems, Health and Community Systems, School of Nursing, University of Pittsburgh, Pittsburgh, PA 15261, United States; School of Computing and Information, University of Pittsburgh, Pittsburgh, PA 15213, United States; Department of Health and Community Systems, Health and Community Systems, School of Nursing, University of Pittsburgh, Pittsburgh, PA 15261, United States; School of Computing and Information, University of Pittsburgh, Pittsburgh, PA 15213, United States; Department of Health and Community Systems, Health and Community Systems, School of Nursing, University of Pittsburgh, Pittsburgh, PA 15261, United States; Department of Biomedical Informatics, University of Pittsburgh, Pittsburgh, PA 15206, United States

**Keywords:** ovarian cancer, information seeking behavior, information avoidance behavior, information sources, interview

## Abstract

**Objectives:**

Despite the importance of using information for ovarian cancer (OvCa) disease management and decision-making, some women with OvCa do not actively seek out information. The purpose of this study is to investigate factors that influence information seeking behaviors and information avoidance behaviors and information resources among women with OvCa and their caregivers.

**Materials and methods:**

We conducted in-depth interviews with OvCa patients or caregivers of OvCa (*n* = 20) and employed deductive and inductive coding methodologies for analysis.

**Results:**

Our analysis revealed 5 emerging themes associated with active information seeking behavior, 5 themes of passive information acquisition, and 4 themes of information avoidance behavior. Additionally, we identified participants’ preferred information sources for OvCa management, such as health organization or government operated resources and web-based social groups.

**Discussion:**

To enhance information access, strategies should be developed to motivate people with OvCa to seek rather than avoid information. The study emphasizes the significance of promoting patient–provider communication and leveraging strong social support networks for effective information acquisition.

**Conclusion:**

Our findings provide valuable implications for clinical practice and policymaking, emphasizing the need to improve access to information for individuals with OvCa. By addressing the identified factors influencing information seeking behaviors, healthcare professionals and policymakers can better support patients and caregivers in their information-seeking journey, ultimately enhancing disease management and decision-making outcomes.

## Background and significance

Ovarian cancer (OvCa, hereafter) is a rare but deadly gynecological cancer.^1^ The 5-year relative survival rate in the United States is 50.8%.^1^ Despite its high mortality and the complexity in managing OvCa, public awareness and educational resources related to OvCa are relatively limited. According to one study, 69% of women with OvCa had never heard of it or knew nothing about it before their diagnosis, and only about 20% of women with OvCa believed they had received all of the information they required at any point.^2^ Moreover, about half of those women reported that their doctor spent <15 min informing and discussing their diagnosis of OvCa.^2^

For individuals with cancer and their family caregivers, successfully managing this complex cancer and treatment requires extensive and targeted information. For example, women with OvCa undergoing treatment suffer from 12 to 14 concurrent symptoms on average.^3^^,^^4^ Women with OvCa are required to gain and learn extensive information to manage the condition, decide on treatment plans, and communicate with providers. Therefore, they may exhibit active information seeking behavior or passive information acquisition behavior. *Active information seeking behavior* refers to individuals actively searching and browsing for information with explicit goals, while *passive information acquisition behavior* refers to individuals passively being exposed to information and absorbing it through monitoring or serendipity.^5–7^ On the other hand, as OvCa is often detected at late stages, when the possibility for cure is unlikely, learning about the unfavorable prognosis of the disease can be very stressful to patients and their caregivers.^8^^,^^9^ Consequently, they may intentionally avoid information, a behavior known as *information avoidance behavior*, which takes place more often in the context of health information seeking.^7^

Previous studies have established the significance of demographic factors (eg, age, gender) and social determinants (eg, income, education) on predicting information seeking and avoidance behaviors of cancer survivors.^10^^,^^11^ However, these studies were primarily cross-sectional and lacked a specific focus on OvCa. Information seeking or avoidance behaviors of individuals with OvCa remain largely under-explored. In addition, factors other than demographic and social determinants, such as individual and contextual factors, have also been less studied.^12^ An in-depth examination of the individual and contextual factors that influence information behaviors from the perspective of women with OvCa is crucial in order to design effective interventions and enhance information access and utilization for women with OvCa.

## Objective

This study aims to investigate the information seeking and avoidance behaviors of individuals with OvCa. Specifically, we aim to answer 2 research questions:

RQ1: What factors are associated with active information seeking, passive information acquisition, and information avoidance behaviors among individuals with OvCa?

RQ2: What are the preferred information sources for individuals with OvCa?

## Methods

### Recruitment

This study is an integral component of the ongoing research project titled Health E-Librarian with Personalized Recommendations (HELPeR) (R01 LM013038). The primary aim of HELPeR is to design a personalized recommendation system for OvCa patients and their caregivers. This system aims to suggest relevant online health information tailored to their needs. To understand their online information-seeking behavior, which is foundational for developing HELPeR, we conducted in-depth interviews with OvCa patients and caregivers. Due to the pandemic, participants were recruited via advertisement on online OvCa communities (eg, NOCC CancerConnect, OvCa related Twitter account) to avoid in-person contact. After expressing interest in the study by contacting research staff, participants were screened via a phone call during which verbal consent was obtained. Our inclusion criteria were: (1) patients with a diagnosis of ovarian, fallopian, or primary peritoneal cancer (any stage) and any time after initial diagnosis, including recurrence OR family caregivers of patients diagnosed with ovarian, fallopian, or primary peritoneal cancer, (2) 18 years or older, (3) ability to read and write in English, and (4) access to computer (or mobile device) and Internet. The study design was approved by the Institutional Review Board of the University of Pittsburgh (IRB #: STUDY19080144).

### Interview process

We conducted in-depth individual interviews with individuals with OvCa. Interview sessions were conducted remotely between May and November 2020 due to the COVID-19 pandemic. A faculty facilitator (YL) and 2 doctoral students (YC and VH) conducted interviews with semi-structured, open-ended questions on participants’ health information seeking or avoidance experience and their perspectives on the information they found. The undergraduate student [HK] observed the interviews and took notes during the interview. Participants were also asked to recall the information sources they had used and to explain the reasons for choosing those specific sources. The interview questions can be found in File S1. Before the interview session, a faculty facilitator provided training to 3 students [YC, YH, and HK] regarding the principles and best practices of conducting interviews. This training included various aspects such as learning to ask open-ended questions, maintaining neutrality, practicing active listening, and taking notes during the interview. Subsequently, the students had the opportunity to observe the faculty facilitator’s initial interviews and actively participate in the interviewing process. Each interview lasted ∼1 h and was recorded with permission. For privacy purposes, all participants used fake names and turned off their video cameras during the interview.

### Interview analysis

All interviews were recorded, de-identified, and transcribed by an external third-party vendor. In this analysis, we excluded responses from the Part 3 questionnaire (File S1) since these answers specifically pertained to a particular aspect of the HELPeR system and were not related to the online health information-seeking behaviors of participants. For both Part 1 and Part 2, we employed both deductive^13^^,^^14^ and inductive^13^ coding methodologies. Initially, a deductive analysis was applied to the transcripts using a predefined set of codes. This was followed by an inductive analysis where new codes emerged organically from the data. To establish our initial code book, the 3 coders and a faculty member (YL) held several meetings. Drawing on existing literature and team discussions, we compiled a list of codes. We then randomly selected 2 transcripts and applied the codes to them, ensuring consistency and acquainting ourselves with the coding procedure. These transcripts were collaboratively reviewed, which allowed us to refine our code book. Subsequently, each coder (YC, VH, and HK) independently analyzed all 20 transcripts using the code book as a reference. When new codes emerged from the transcripts, a meeting was convened to discuss their incorporation. Coders grouped these codes into overarching categories and subcategories, developing themes, and met weekly or bi-weekly to discuss agreements and discrepancies. If the coders disagreed on any aspect of coding, the faculty facilitator, YL, who possesses extensive expertise in qualitative methodology, resolved these differences. We utilized NVivo, a qualitative data analysis software, to streamline the code sorting and augment the manual coding process.^15^

## Results

### Overview

A total of 20 individuals were recruited and completed interviews. Eighteen participants were women with OvCa, and 2 were family caregivers of OvCa patients. The average age of participants was 58, and 70% of participants (*n* = 14, 70%) were diagnosed with advanced OvCa (stage III or IV). Half of the participants had finished initial treatment and were in remission (*n* = 10, 50%). Table 1 presents the demographic information of the participants. Table 2 presents the themes identified in this study. The subsequent section provides comprehensive descriptions and explanations for each theme.

**Table 1.** Participant demographics.

Variable(s)	Number of participants (*n* = 20, %)
Role	
Patient	18 (90)
Caregiver	2 (10)
Gender	
Male	1 (5)
Female	19 (95)
Race	
White	20 (100)
Black	0
Other(s)	0
Employment	
Currently working	7 (35)
Not currently working	11 (55)
Prefer not to answer	2 (10)
Marital status	
Married/living with partners	13 (65)
Not living with partners	6 (30)
Prefer not to answer	1 (5)
Education level	
Highschool graduate	2 (10)
College graduate	11 (55)
Graduate or professional school	5 (25)
Prefer not to answer	2 (10)
Age	58.3 (Range: 40-75, SD: 9.0)
Cancer information	
Cancer stage	
I	4 (20)
II	0
III	7 (35)
IV	7 (35)
Reported unclear	2 (10)
Treatment received	
Surgery	18 (90)
Chemotherapy	19 (95)
Radiation	1 (5)
Immunotherapy	3 (15)
Targeted therapy	2 (10)
Disease trajectory	
Currently receiving chemotherapy for initial diagnosis	5 (25)
Finished initial treatment and/or NED	10 (50)
Recurrence	3 (15)
Currently enrolled in clinical trial	2 (10)

**Table 2.** Overview of themes associated with information seeking and avoidance behaviors.

Themes associated with active information seeking	Themes associated with passive information acquisition	Themes associated with information avoidance
Filling knowledge gaps Seeking OvCa survivor stories To reduce anxiety and uncertainties Being unsatisfied with communication with providers To gain confidence	Having sustained information resources Having personal connections with health professionals Having personal connections with cancer survivors Being satisfied with communication with providers Having trust in providers’ knowledge	Lacking knowledge to understand the information Unable to evaluate the relevance and quality of the information Fear of exposure to negative information and emotion Feeling powerless about cancer curability

### Themes associated with active information seeking

All but one participant (P15) reported that they had experience actively seeking information for OvCa (*n* = 19/20, 95%). Five themes related to active information seeking were identified:

#### Filling knowledge gaps

Participants indicated that they would consciously proceed to seek information when they identified a gap in their knowledge. For example, P16 said: “*my platelets were too low to get chemo, and I looked up and saw what kind of foods you could eat to help bring it up.*” The participants also reported that they could recognize a knowledge gap in many scenarios, and unknown medical terminology about OvCa was reported as a common trigger of active information seeking, as P08 said: “*When I got the original test results on MyChart, there was a bunch of words that I wasn’t really sure what it meant. So, I looked up the words and it said that it was an ovarian tumor.*” Similarly, P17 would use online encyclopedias, like Wikipedia, to look up medical terms: “*if I don’t understand something, a lot of times I’ll kind of go to Wikipedia and try to look up a term or try to look up.*”

#### Seeking OvCa survivor stories

Several participants reported that they sought and read stories from other OvCa survivors, especially from online social groups, to get knowledge, hope and inspiration. P12 learned about treatment information from survivor stories, “*Reading people’s stories was truly important to me because you could see that their doctor may have taken a different approach. It created questions for me, which was good, that I could go back and question Dr XX about.*” P18 told us how she sought survivor stories from peers on Facebook and benefited from them: “*When somebody tells you their story, you can pick up their strength and maybe improve your own strengths. It’s a tough fight.*”

#### To reduce anxiety and uncertainties

Participants identified information seeking as a way to reduce anxiety and uncertainties and set their minds at ease. For example, P11 said that she sought information to reduce her anxiety associated with cancer recurrence: “*for me, right now the anxiety is tied to preventing cancer from recurring. So the knowledge of what to do is kind of freeing to reduce the anxiety. So I need the knowledge and the confidence that I know what to do.*” P04 is a family caregiver for her sister, and she told us that her sister looked up information to deal with uncertainty associated with OvCa: “*Obviously, ovarian cancers are a hard one because there are some people who live 15, 20 years with ovarian cancer or get cured, and there’s others that aren’t that fortunate. So I think one of the things with ovarian cancer is dealing with that uncertainty. My sister wants to have hope, but she also wants to be realistic in planning for her children’s future and understanding if there’s—maybe when the right time to seek palliative care is versus putting her body through something that is going to be so detrimental to her quality of life and probably won’t extend it.*”

#### Being unsatisfied with communication with providers

Some participants shared perceptions that their healthcare providers did not provide sufficient transparency regarding treatment information, leading them to actively seek out information on their own. For example, P05 reported that “*It’s like everybody’s in the dark. The doctors don’t tell you what they’re going to give you in advance, and we’ll worry about everything later. I don’t believe that should be the way it should be.*” She also explained why she thought it was important to seek and learn information proactively instead of being passive about clinical trials “*when it comes to clinical trials and you building up exclusion conditions, that you may not be able to get into clinical trials because you’ve got the wrong treatment or you chose a treatment that you shouldn’t have if you definitely knew that it would exclude you from a clinical trial.*” P03 reported that the lack of communication in her treatment was one of the driving factors that prompted her to independently seek out information: “*you are just pushed on the treatment train, like this is what you do. And one of my doctors said, “You’re crazy if you don’t do this. You must do this.” They tell you, you have to do it, but they don’t explain why or what the alternatives are, or whatever. That’s not enough. I need to know why I need to do it. What’s going to happen if I don’t.*”

#### To gain confidence

Participants said that collecting as much information as possible and getting involved in the treatment and decision-making process would help them feel more confident of the treatment they received. P14 said: “*I’m not ignorant of the fact that it [reoccurrence] can pop up in some other form. But I got much confidence to keep everything in my hand.*” P03 even said that she felt the nurse who told her not to look up information on Google was “patronizing and insulting”: “*one nurse said to me, “Don’t go on Dr Google,” and I thought that was patronizing because I’m like, “I am definitely going to be trying to research and understand this disease.” I didn’t think that was helpful. I thought that was a little bit insulting.*”

### Themes associated with passive information acquisition

Thirteen participants reported how and why they obtained OvCa-related information passively in the interviews (*n* = 13/20, 65%). Five themes were identified:

#### Having sustained information resources

Participants reported using routine information resources to passively receive OvCa-related information regularly. For example, P5 said: “*I have a couple of websites that will kind of give me a daily email update saying, ‘Okay, this is what just came down the pipe.’*” Besides, participants also told us that they occasionally attended the classes or seminars provided by hospitals and encountered information on a range of topics. P17 shared: “*UPMC used to have a Survivor series, and they would have seminars or classes or meetings about certain things, subject lines…That’s pretty much been my main sort of knowledge or information. And it could range from—chemo-brain, to nutrition, to anxiety and the mental—from a psychologist or psychiatrist.*” Likewise, P20 said: “*I go to Dana-Farber. The Zakim Center has tons of classes.*” In addition, sustained use of online health communities would also allow participants to acquire information and construct knowledge unconsciously. For example, P10 had been using the Facebook group for OvCa for years, and she commented that: “*I’ve gotten a lot of information from them [Facebook group] as to what to expect when I first was diagnosed that I feel confident in my doctors and my nurses with my treatment.*”

#### Having personal connections with health professionals

Five participants reported having a personal connection to a healthcare professional within their social network, who they relied on for information regarding their cancer. These professionals could be their family members, friends, or neighbors. The participants asked professionals for help to seek information for them or asked for referral in gynecological cancers. For example, when P11 talked about her primary information source, she said: “*I relied on my brother-in-law, a general doctor, and he researched the top hospital for cancer then the gynecology department and then found the chairman of the gynecology department.*”

#### Having personal connections with cancer survivors

People who have had cancer in the patients’ personal network is another supportive information resource that allows our participants to be passively exposed to OvCa-related information. For example, P14 commented: “*I have a couple of good friends that have been cancer survivors and gone through it that have been good mentors for me.*”

#### Being satisfied with communication with providers

The participants who reported a high level of satisfaction with their communication with their healthcare providers tended to passively absorb information from them. They felt satisfied and eased when the providers explained information and decisions to them face-to-face patiently and respectfully. P16 said: “*He’ll [doctor] sit down and explain everything, and if I don’t understand it, he’ll go back over it, and they may give me a paper to tell me what the side effects of the chemo is and everything. And the doctor asks for my opinion, what I think, and he is wonderful.*”

#### Having trust in providers’ knowledge

Participants who trusted their providers and believed that “*they know better than anybody*” would tend to be passive information recipients and more likely to rely on providers to tell them what to do or what not to do. P17 said: “*That would be my point of preference, just to receive the information from a provider or someone that’s working on something specific to that topic.*” One participant (P20) attributed her trust to her doctors to her distrust in her personal knowledge: “*I am not comfortable with my science knowledge to ever suggest Dr YY. I have to trust him.*”

### Themes associated with information avoidance

Four participants (20%) reported avoiding information about their cancer, and among them, one participant tried to avoid any form of active or passive information acquisition. Four themes related to information avoidance emerged from the interviews:

#### Lacking knowledge to understand the information

The participants reported that they may choose to consciously ignore the knowledge gap and avoid information if they felt overwhelmed by the information. For example, P13 commented: “*Just layman terms are great with me. If it gets too complicated, then I get frustrated, and then I get more confused, and then I have to look up more words. And by the time I’m done, I’m getting totally upset.*” Insufficient search expertise could also impede participants from seeking information themselves especially from some online sources, such as digital databases. For example, P05 said she avoided using Cochrane Library, because “*it’s so difficult to search in. I tried to go on there, and I have no clue how their search works. It just shows me that they have a million and a half studies.*”

#### Unable to evaluate the relevance and quality of the information

A number of participants expressed a lack of confidence in their ability to assess the relevance and quality of the information they sought, particularly information obtained from the internet. As a result, they preferred to avoid information. The participants also reported a large amount of irrelevant information, which further contributed to their aversion to seeking information. For example, P17 said: “*I’m a little leery about reading things—it’s hard to tell sometimes what’s drug-pushed articles and what’s actual medical exam articles. And I get nervous about not knowing whether or not this relates to my specific process in my journey.*” Also, P11 said, “*what I initially read online was really frightening and wasn’t necessarily true.*”

#### Fear of exposure to negative information and emotion

Some participants reported that exposure to negative information and emotions could also push them away from an information source permanently. P17 retreated from online social groups and commented: “*it would actually make me more sad to be there at times because we were constantly getting new people, which was great, but it was constant reminders of the negative where I’m trying to just move forward with the positive and not have to be reminded every single time I’m there. And so, I did kind of move away from the group and gave myself some space.*” P15 also blamed the online community: “*they always sound worse than what I had felt. I have not been—I mean, I’ve been sick, but I have not been like what I’ve heard.*” She also added: “*I find I get depressed and more anxious if I read the bad.*”

#### Feeling powerless about cancer curability

P13 told us about her overwhelming feelings toward the information from the American Cancer Society, an authoritative cancer source: “*Everything’s negative there. It says you have a very low chance of living. Ovarian cancer is one of the worst. They give you absolutely no hope. It scares me. That site is one of the main reasons why I stopped looking stuff up.*” Participants who believed that they had no control over their disease and were uncertain about the cancer curability chose to avoid information. P15 told us “*I guess there’s the test that basically tells them [doctors], in the blood test, about the amount of cancer maybe in you… I’m trying not to be angry, blame people, or blame anything. Maybe I’m one that puts my head in the sand… I quit reading the results of my tests.*”

### Preferred information sources

We categorized the information sources utilized by participants into 11 categories and counted the number of participants who reported using each type of source for active information seeking or passive information acquisition (Table 3). All participants, except for participant P15 who avoided any form of OvCa-related information, reported obtaining information through active or passive means from various types of information sources.

**Table 3.** Information sources reported for active information seeking and passive acquisition.

Category	Sources reported	Number of participants who reported using the source for active information seeking	Number of participants who reported using the source for passive information acquisition
Health organization or government operated resource	American Cancer Society (ACS), National Institutes of Health (NIH), National Comprehensive Cancer Network (NCCN), ClinicalTrials, MedlinePlus, Friends of the Oncology and Radiotherapy Centre, Exeter (FORCE), Imerman Angels, Gilda’s Club, Clearity Foundation, Sager Strong Foundation	13	0
Web-based social group	Facebook, Twitter, National Ovarian Cancer Coalition, Sisterhood of Ovarian Cancer Survivors, CaringBridge.com, inspire, Smartpatient.com	11	2
Literature or book	Google Scholar, PubMed, Cochrane Library, OvCa-related book	7	0
Healthcare provider	Doctor, nurse practitioners, oncologist, surgeon, nutritionist, patient advocate	6	7
Hospital-based or university-based resource	Mayo Clinic, University of Pittsburgh Medical Center, Gustave Roussy, Northwestern.edu, the Baptist Hospital, Dana-Farber classes, Johns Hopkins, Mass General, Harvard, MD Anderson or Sloan Kettering, University of California at San Francisco	4	4
Personal network	Family, friends, relatives,	2	5
Video and audio	YouTube, OncLive, podcasts	2	2
General health information and updates	Cure Today, Wikipedia	2	1
Mobile app	Noom, MyNet	1	0
Local support group	Local support group for women with ovarian cancer	1	0
E-health portal	MyChart, MyUPMC	0	1

In terms of reasons of choosing an information source, participants indicated that they considered trustworthiness and authoritativeness of the sources. For example, P12 commented that: “*It has to be one of the big names. When I look for information now, I usually try to look for hospitals that have information resources and then from there I usually go to direct research.*” Notably, the NCCN guidelines developed by the National Comprehensive Cancer Network were highly valued by our participants for its comprehensiveness and understandability. For example, P02 said, “*That [the NCCN guidelines] is something, I think, should be in the hands immediately after a woman is diagnosed with ovarian cancer. I think that is the most comprehensive and easily understandable document I’ve seen.*”

Participants also appreciated the diversity of the content, up-to-date information, and the social support exchanged in the groups; thus, they preferred to join online social groups. For example, P05 said, “*it’s kind of like shopping on eBay. You can shop worldwide.*” Similarly, P03 said: “*you can kind of see the different questions people have when they’re diagnosed, in treatment, the clinical trials, genetic testing, lifestyle factors, sort of a bunch of different things that people go through.*” In addition, P10 said that she actively sought information from web-based social groups instead of search engines because the information was more up to date: “*from searching on Facebook… I seem to find the ones like googling those to be kind of outdated.*” Moreover, participants commented that they received various informational and emotional support from peers in web-based social groups. For example, P08 told us: “*it was definitely helpful to know other people that have had that same experience and how easy or how hard it was.*”

## Discussion

### Principal findings

Our in-depth interviews revealed comprehensive information about the factors associated with information seeking behaviors and information avoidance behaviors as well as preferences of information sources among individuals with OvCa.

First, this study found that participants’ prior knowledge of OvCa plays a critical role in determining their information seeking behavior. Several participants stated that they actively sought information when they recognized a gap in their existing knowledge. This finding aligns with established information-seeking theories, such as Dervin’s sense-making theory^16^ and Belkin’s notion of anomalous state of knowledge,^17^ which posit that filling knowledge gaps drives active information seeking. Given that OvCa is a complex disease that requires a high level of knowledge for symptom management and treatment, participants actively or passively sought information to fill gaps in their knowledge.

Notably, our study extends the literature of knowledge gap and information seeking by highlighting that knowledge gap could also lead to information avoidance, an extremely opposite outcome, in the context of OvCa. The major difference may stem from the extent of the knowledge gap and the presence of anxiety and information overload. Participants who actively sought information primarily identified gaps in medical jargon or less medical-related knowledge. On the other hand, those who chose to avoid information had larger gaps, often accompanied by information overload and overwhelming emotions or frustrations during the knowledge acquisition process. Due to the low prevalence of OvCa, information about the disease is generally scarce and of lower quality.^18^^,^^19^ These findings suggest that the information provided to individuals with OvCa, especially when first diagnosed, should be more intuitive and easily understandable to reduce the steep learning curve associated with this complex disease.

Second, our findings corroborate prior research by providing additional evidence that fear of negative information and emotions, along with a sense of hopelessness, can contribute to information avoidance.^20–22^ The findings align with previous research by Miles et al,^20^ which demonstrated that higher cancer fear can predict increased cancer information avoidance, mediated by perceived severity. Women with OvCa experience high rates of recurrence, high symptom burden, and high intensity of treatment,^23^ which all could lead to extreme anxiety and uncertainty. Our participants mentioned that they were often frightened by negative information or stories, such as dramatically worsening conditions or death. Our findings stressed the importance of developing strategies to reduce cancer fear and build confidence in cancer curability for people with OvCa so that they can confront this deadliest gynecological cancer. For instance, future cancer education materials and online content should be written in a neutral tone to avoid arousing fear and inform individuals about the possibility of a cure.

Third, our findings underscore the significance of patient–provider communication and patient satisfaction in influencing information-seeking behaviors. Healthcare providers were a primary information source for both active and passive information seekers among our participants. In particular, trust in a doctor’s expertise in OvCa and satisfaction with communication with doctors motivated these individuals to rely on their providers as their main source of information. Contrarily, if individuals felt they were not adequately involved in the communication, they would be less satisfied and more likely to question the information provided by their providers. The findings align with previous literature, where participants emphasized the importance of compassionate and accessible care providers in facilitating their reliance on them for treatment information.^12^^,^^24^ Furthermore, our results underscored the significance of patient self-efficacy in decision-making. Participants with high self-efficacy valued being involved in decision-making about their treatment plan and expected transparency from their providers about their disease and treatment options. They thought that they should not only actively seek information, but also be a “*partner*” of the doctors and participate in every decision-making about her treatment plan. These insights highlight the need for empowering patients with OvCa to play an active role in their care decisions.

Fourth, powerful social support is a significant enabler of passive information acquisition for people with OvCa. This finding aligns with Johnson’s Cancer-Related Information Seeking model,^5^ which highlights the significance of one’s present social network in influencing information-seeking outcomes. In general, a strong support system, either from providers or people they know in person, or the participation in a sustained online support group, enabled the participants to passively rely on the information they acquired from others, instead of actively seeking the information on their own. In line with previous work,^25^^,^^26^ we found that web-based social groups, such as online health communities and social networking sites, are playing leading roles in supporting people with OvCa’s active information seeking, while on the contrary, only one participant reported attending in-person support groups. This might be because the current study was conducted during the Covid-19 pandemic when the participants might be concerned to join in-person support groups. Moreover, we found that the participants obtain information via multi-devices and in multi-formats, including videos, audios, mobile applications, and E-health portals, although the sample size was small and thus, further research is needed to examine the usage and characteristics of these information sources.

Lastly, our study highlights the significance of health organization or government-operated resources, such as NCCN guidelines, as preferred sources for active information seeking among individuals with OvCa. These resources were also actively shared within online health communities, as demonstrated in a previous study.^26^ Participants in our study attributed their preferences to the trustworthiness and authoritative characteristics of these sources. In terms of passive information acquisition, it is noteworthy that healthcare providers and personal networks emerged as the most preferred resources. The unique benefits of these resources, including trust and familiarity, tailored information, and emotional connections, might contribute to people’s preferences for passively acquiring information from them. By shedding light on the role of various information sources in supporting individuals with OvCa’s information-seeking behaviors, our research makes a significant contribution to the broader literature on cancer-related information seeking and emphasizes the importance of fostering and leveraging different channels to aid people with cancers in their information acquisition journey and further improve their overall cancer care experience.

### Implications

Future clinical practice and policy making should consider the implications of this study. The findings underscore the need to improve patient–provider communication and enhance patient involvement in decision-making processes to better inform individuals with OvCa. Clinical interventions could focus on increasing patients’ understanding of their disease-related information, which can improve confidence and self-efficacy in communicating with healthcare providers. They should also promote patients’ participation in support groups, both online and in-person, to strengthen the social support system. Healthcare providers could undergo training to enhance their communication skills. Implementing patient-centered communication approaches, such as active listening and shared decision-making, can foster better information exchange and increase patient satisfaction.^27^ Additionally, healthcare facilities can utilize digital interventions, such as patient portals and telemedicine platforms, to facilitate easy access to relevant information and encourage ongoing communication between patients and providers.^28^ Furthermore, given that health care providers are primary sources for both active and passive information seeking, healthcare providers could help promote awareness of the potential curability of OvCa and distribute education resources to reduce patients’ anxiety and fear and encourage them to take an active role in their treatment plan.

On the other hand, policymakers should focus on developing comprehensive education materials, such as brochures, videos, and mobile applications, to provide information about OvCa that is easy to understand and accessible to everyone. Design principles that enhance readability should be considered, such as using plain language and intuitive visual aids and formatting for better comprehension.^29^^,^^30^ Efforts should also be devoted to improving information literacy among people with OvCa and their caregivers to empower them to make informed decisions. Strategies could include implementing community outreach initiatives (eg, campaigns, workshops) aimed at reaching vulnerable, marginalized populations with low literacy levels.

### Limitations

The study has several limitations. First, the sample was relatively homogeneous despite efforts to recruit a diverse group. Most participants were White females (except for one male caregiver) and highly educated, which may limit the generalizability of the results to the broader OvCa population. This homogeneity may be explained by the fact that White women have the highest incidence rate of OvCa.^31^ Additionally, our online recruitment might have constrained the diversity of our sample due to the digital divide within the online OvCa communities from which we recruited participants.^32^ Furthermore, it is important to acknowledge the potential impact of the COVID-19 pandemic on participant recruitment. However, it is worth noting the significant racial/ethnic disparities in OvCa. The Surveillance Epidemiology and End Results (SEER) program reports a higher incidence of epithelial OvCa among White women, while African-American women tend to experience worse survival outcomes.^33^^,^^34^ These disparities underscore the critical need for a representative population in OvCa research, ensuring that the perspectives and experiences of diverse racial and ethnic groups are adequately captured. Second, the sample size of the interview was relatively small, which could also impact the generalizability of the study. Third, the obtrusive methods, such as interviews, may not reflect the participants’ actual practice of source selection in natural settings because the participants were aware of being studied and may have tried to behave in a favorable way. In addition, we acknowledge the potential influence of recall bias in the interview, as participants were asked to recall the information sources they had used. This may lead to discrepancies between reported information sources and actual practice, as participants may not accurately remember or disclose all the sources utilized. Future research is anticipated to play a pivotal role in addressing these limitations and enhancing the accessibility of information and knowledge resources for individuals with OvCa. The high mortality rate of OvCa underlines the urgency of this endeavor.^1^ To this end, future research should work on enhancing sample diversity through targeted recruitment strategies and collaborations with diverse communities. It would be beneficial to explore hybrid recruitment approaches that overcome the constraints of online recruitment. Increasing the sample size and employing a mixed-methods approach could potentially further improve the generalizability of findings and provide a more comprehensive understanding of women with OvCa’s information-seeking behaviors in natural settings.

## Conclusion

Despite the significant impact of OvCa on women’s health, there is a lack of research on how to meet the information needs of women with OvCa. Our study provided perceptions of 18 women with OvCa and 2 family caregivers on their health information seeking behaviors, focusing on their self-reported factors of active information seeking, passive information acquisition, and information avoidance. Furthermore, this is the first study to identify their preferred information sources of active information seeking and passive information acquisition. Our findings suggest that reducing the learning curve, alleviating fear, and promoting cancer curability can encourage people with OvCa to seek information more actively. We also highlight the importance of improving patient–provider communication and social support, in addition to developing education materials. However, considering the relatively small sample size and limited diversity among our study participants, these findings may not be generalizable to all women with OvCa and their caregivers. As such, further in-depth studies involving various groups of OvCa patients and caregivers are needed. These groups should represent a wide range of socio-demographic backgrounds and span across all stages of the cancer trajectory to ensure a comprehensive understanding.

## Supplementary Material

ooae011_Supplementary_Data

## Data Availability

The data underlying this article will be shared on reasonable request to the corresponding author.
