# Supplementary material for: Women with ovarian cancer’s information seeking and avoidance behaviors: an interview study
Source: JAMIA Open. 2024 Feb 21;7(1):ooae011. doi: 10.1093/jamiaopen/ooae011 (PMC10881099; doi:10.1093/jamiaopen/ooae011)
Supplement: ooae011_Supplementary_Data [file ooae011_supplementary_data.pdf]

Semi-Structured Interview Protocol

**Part 1: Background Information**	
**Purpose**	**Verbal protocol**
Condition background	1. When were you diagnosed with ovarian cancer?<br>2. What stage was the cancer when you were diagnosed?<br>3. What treatments have you received: (check all that apply – surgery, chemotherapy, radiation therapy, immune therapy, hormone therapy)<br>4. What is the status of the cancer now? Currently receiving treatment for my initial diagnosis; Finished initial treatment and no current evidence of disease; on treatment for a recurrence; other<br>5. Do you live with family? If so, who would that be? Who in the family are helping you? Do you have another caregiver? If so, who is the caregiver? How often does the caregiver help you?
Knowledge level background	6. How would you say that your overall knowledge about the ovarian cancer is… (Likert scale 0-10, 0: No knowledge on this topic, 10: Expert level of knowledge)
**Part 2: Previous Search Experience/Current Practice**	
**Purpose**	**Verbal protocol**
Information need	7. What are one or more topics that you would want to know more about?<br>8. Why do you want to know about these topics?
Knowledge level	9. How would you say that your current knowledge about *topic XXX [answer of the Q6]* is… (Likert scale 0-10, 0: No knowledge on this topic, 10: Expert level of knowledge)
Search strategy	10. Generally, how do you find information for topic XXX? Can you describe in details the steps you would take to find the information?<br>- *Prompts: do you search on your own or someone else will search for you?*<br>11. Do you have particular sources that you go to get information about cancer? How did you learn about these sources? Were they useful? Why or why not?

	12. How do you judge whether the information is relevant/important/trustworthy or not? What criteria do you use to judge relevance?
Outcome	13. How do you plan to use the information you get about topic XXX?
**Part 3: Perspectives on the Recommendations**	
**Purpose**	**Verbal protocol**
Perspectives	We have selected online information that seems helpful for you. Could you take a look and let us know if the recommended information meets your needs?<br>14. Could you tell us whether this suggested information is helpful/useful for you?<br>15. Why do you think it is useful/ not useful to you?<br>16. Is the recommendation *relevant* to your current situation? Could you explain how the recommendation is relevant to your current situation?<br>17. Would you recommend this information to others with the same problem?<br>18. How would you say the knowledge level in this recommended article is… (Likert scale 0-10, 0: No knowledge on this topic, 10: Expert level of knowledge)<br>19. Do you think the recommended information is too hard or too easy for you? Or just at the right level? Why?
Outcome	20. How often would you want to get follow-up recommendations?<br>- *Prompts: any time when there is a new relevant information, every time they come for a treatment, once every month, or others*<br>21. Do you think you would use the recommendation in your daily life?<br>22. Could you explain how you will use the information to manage your cancer?
